# Chagas Disease and Domestic Medical Screening Guidance for Newly Arrived Individuals Under a Humanitarian-Based Immigration Status: A Call for Action

**DOI:** 10.4269/ajtmh.22-0309

**Published:** 2022-11-14

**Authors:** Nelson Iván Agudelo Higuita, Carlos Franco-Paredes, Andrés F. Henao-Martínez, Norman L. Beatty, Jennifer Manne-Goehler, Colin J. Forsyth

**Affiliations:** ^1^ Department of Medicine, Section of Infectious Diseases, University of Oklahoma Health Sciences Center, Oklahoma City, Oklahoma;; ^2^ Hospital Infantil de México, Federico Gómez, Mexico City, Mexico;; ^3^ Department of Medicine, Division of Infectious Diseases, University of Colorado Anschutz Medical Campus, Aurora, Colorado;; ^4^ Department of Medicine, Division of Infectious Diseases and Global Medicine, University of Florida College of Medicine, Gainesville, Florida;; ^5^ Division of Infectious Diseases, Brigham and Women’s Hospital, Harvard Medical School, Boston, Massachusetts;; ^6^ Drugs for Neglected Diseases Initiative–North America, New York, New York

## Abstract

Chagas disease is considered one of the most important neglected tropical diseases in the Western Hemisphere, given its morbidity, mortality, and societal and economic burden. The United States has the fifth highest global burden of Chagas disease. Every year, thousands of migrant people from Latin America and throughout the globe travel to the U.S.– Mexico border searching for asylum. The U.S. CDC’s *Guidance for the U.S. Domestic Medical Examination for Newly Arriving Refugee*s provides recommendations to safeguard the health of individuals who enter the United States with a humanitarian-based immigration status as defined by the CDC’s guidance under Key Considerations and Best Practices. We encourage the inclusion of *Trypanosoma cruzi* infection screening recommendations in this guidance as an important step toward understanding the risk and burden of Chagas disease in this vulnerable population, strengthening their access to care and contributing to the 2030 objectives of the WHO’s neglected tropical diseases road map.

The U.S. CDC first published *Guidance for the U.S. Domestic Medical Examination for Newly Arriving Refugees *(formerly known as *Domestic Medical Screening Guidelines*) in 2006.^1^ The document provides health-care professionals with evidence-based recommendations to safeguard the health of individuals entering the United States with a humanitarian-based immigration status as defined by the CDC’s guidance under Key Considerations and Best Practices. The guidance emphasizes prevention, prompt identification, and treatment of communicable and noncommunicable diseases of individual and public health importance. The document includes disease-specific aspects of tuberculosis, malaria, Hansen’s disease, soil-transmitted helminthiasis, strongyloidiasis, schistosomiasis, hepatitis B and C, and sexually transmitted infections such as syphilis, HIV, gonorrhea, and *Chlamydia*.^1^ The guidance lacks a screening recommendation for *Trypanosoma cruzi* infection—the most important parasitic disease in the Western Hemisphere based on its morbidity, mortality, and societal and economic burden.^2^

A substantial number of individuals from Chagas disease–endemic areas seek entry to the United States with a humanitarian-based immigration status and are eligible for comprehensive domestic medical screening. In 2019, approximately 34.5% of the 46,508 individuals granted asylum originated from Latin American countries endemic for Chagas disease.^3^ An estimated 6 to 7 million people have left Venezuela since 2014, and Venezuelans are now the fifth-largest South American immigrant population in the United States.^4^^,^^5^ Worldwide, there are currently 470,000 refugees and asylum seekers from Central America.^6^ Because migration to the United States is an increasingly global and transcontinental phenomenon, migrants from various Latin American, Caribbean, Asian, and African countries travel circuitous routes through the rainforests and other ecosystems of South and Central America bound for the U.S.–Mexico border.^7^

Asylum seekers and other migrant populations traveling within Latin America are at risk for vectorial transmission of *T. cruzi* infection, given their route of travel and living conditions during their journey. Before reaching the U.S.–Mexico border, and depending on the country of origin, migrants may travel through the Gran Chaco in the Southern Cone,^8^ the Darien Gap located between Colombia and Panama,^9^ and sections of southern Mexico within the states of Chiapas, Jalisco, Oaxaca, and the Yucatan Peninsula.^10^ More than 20 triatomine species naturally found within these regions are known to harbor *T. cruzi*, including well-known Chagas disease vectors: *Triatoma dimidiata*, *Triatoma infestans*, *Panstrongylus geniculatus*, *Rhodnius prolixus,* and *Rhodnius pallescens*.

One evaluation found 79 of 392 migrants at the Mexico–Guatemala border had seen a triatomine in places they had slept during their travel. Of this cohort, 12 individuals (3.1%) had serological evidence of Chagas disease by two separate ELISAs.^11^ Migrants with Chagas disease were more likely to have been born in a rural setting or lived in a house with the roof, walls, or floors made of nylon, plastic, or cardboard, or to have slept outdoors.^11^ Another investigation among Guatemalan, Salvadorian, Honduran, and Mexican migrants traveling through Mexico en route to the United States or Canada found that 20% (24 of 120) had serological evidence of Chagas disease. The majority (86%) recognized the triatomine, and 62 individuals (59%) reported being bitten.^12^ In Spain, for example, an estimated 6% of Latin American migrants are infected with *T. cruzi*, with vertical transmission occurring in 3 of 100 live births.^13^

Refugees and asylum seekers may spend months in transit countries in Central and South America while preparing for the next phase of their journey or, frequently, as victims of extortion or kidnapping.^14^^–^^16^ These travelers often have to wait at U.S. border crossings as immigration applications are processed. The U.S. Department of Homeland Security’s 2018 Migration Protection Policy forced those seeking entry into the United States to await immigration proceedings in Mexico.^17^ This migrant population is relegated to crowded, ad hoc camps or the streets of border towns with known triatomine vectors.^18^ Thus, even migrants from nonendemic countries in the Caribbean, Asia, and Africa—currently about 10% of migrants stranded in Mexico^16^—may meet current Chagas disease screening criteria by having lived more than 6 months in an endemic country.^19^

Foodborne transmission and orally acquired *T. cruzi* infection during the migratory journey poses another risk.^20^^,^^21^ Ingestion of food or beverages contaminated with triatomines or their metacyclic trypomastigote-containing feces has caused outbreaks of orally acquired Chagas disease in many Latin American settings.^22^^–^^24^ Most cases occur after ingesting unpasteurized homemade juices of plant origin, such as sugar cane juice, palm wine, açai berry, or wild-animal meat.^22^^,^^25^ Because development of infective *T. cruzi* metacyclic trypomastigotes occurs in odoriferous anal glands of opossums, *T. cruzi* can also be transmitted from the ingestion of opossum meat or blood.^26^ Alternatively, opossums or armadillos may contaminate food prepared for human consumption, given their ability to aerosolize secretions from the anal glands.^20^^,^^26^ Food insecurity during migration through the rainforests and rural areas of South and Central America, and Mexico results in unsanitary food practices. Implementing preventive measures is a major challenge, making screening even more important.^20^

In contrast to vector-borne *T. cruzi* transmission, the oral route entails a shorter incubation period and more severe clinical manifestations, including severe cardiac involvement.^22^^,^^24^^,^^25^ The high attack rate during foodborne outbreaks is likely a result of the ingestion of a greater parasitic load.^24^^,^^26^ Clinicians should suspect possible acute orally acquired *T. cruzi* infection when individuals present with undifferentiated febrile syndromes associated with bilateral palpebral edema, facial edema, and lower extremity edema with or without manifestations of cardiac involvement.^25^ These clinical signs are essential clues in the differential diagnosis of acute febrile syndromes in recently arrived migrants to the United States. The use of thick and thin smears of peripheral blood offers the greatest sensitivity and positive predictive value to detect parasitemia during acute infection.^22^^–^^24^

Importantly, screening for *T. cruzi* infection in women can address the risk of congenital transmission, an important route of infection in Latin America and the main transmission route in nonendemic countries, including the United States. In Latin America, vertical transmission accounted for 22% of all cases in 2010, with the highest estimated annual number reported in Mexico, Argentina, Colombia, and Venezuela.^27^ A recent systematic review and meta-analysis that studied the frequency of vertical transmission in pregnant women living outside Latin America found an overall seroprevalence of *T. cruzi* infection of 4.6% and a global congenital transmission rate of 3.5%.^28^ Although only a few cases of congenital Chagas disease have been documented in the United States,^29^^,^^30^ there are an estimated 63 to 315 annual congenital infections that are not identified as a result of the lack of a screening program.^31^

Screening migrants who may have acquired *T. cruzi* infection prior to or during migration represents an opportunity to provide timely treatment. Anti-trypanosomal therapy is effective during acute and early chronic forms of Chagas disease.^32^ However, untreated Chagas disease can lead to a 5% annual risk of chronic cardiomyopathy.^33^ Early identification of congenitally infected neonates is important because the treatment success rate surpasses 90% in this patient population.^34^ In the United States, screening for congenital Chagas disease is cost-effective for rates of congenital transmission ≥ 0.001% and for all levels of maternal prevalence ≥ 0.06%.^35^

Despite strong progress in the control of Chagas disease in Latin America, the United States is currently home to more people with *T. cruzi* infection (> 300,000) than 16 of 21 Latin American endemic countries.^27^^,^^36^ Nonetheless, < 1% of those with the disease have accessed diagnosis or treatment because of numerous barriers, and health-care providers are often unaware of who is at risk.^37^^–^^39^ Although screening for *T. cruzi* infection with serological assays is a critical U.S. public health intervention among immigrants from Chagas-endemic settings, there is an urgent need to consider screening recently arrived refugees, asylum seekers, and other migrant persons, including extracontinental migrants from nonendemic countries in Africa, Asia, and the Caribbean who have spent prolonged periods in highly endemic areas in Latin America (Figure 1). Nonendemic countries with fewer *T. cruzi*–infected individuals than the United States have included recommendations for *T. cruzi* screening in their national guidelines,^13^ and recommendations for screening and diagnosis of Chagas disease in the United States were recently published.^19^ We urge consideration of Chagas disease in the* Guidance for the U.S. Domestic Medical Examination for Newly Arriving Refugees*. This could provide an important gateway to understanding more fully the risk and burden of this neglected disease in a vulnerable population that faces unique health-care access challenges and leads to more widespread screening of high-risk groups beyond this context. Moreover, ensuring timely diagnosis and treatment can help prevent future congenital transmission and progression to more severe disease. Ultimately, a comprehensive public health approach in which screening and care is available to all people at risk of Chagas disease is key to achieving the goals in the WHO’s 2030 neglected tropical diseases road map.^40^

**Figure 1.**
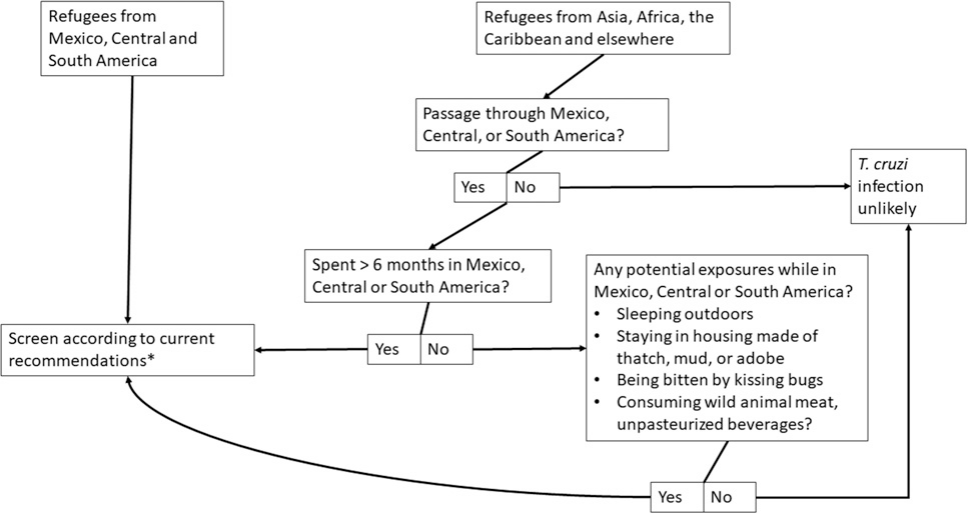
Algorithm for screening and diagnostic confirmation of *Trypanosoma cruzi* infection in individuals that enter the United States under humanitarian-based immigration status. Humanitarian-based immigration status applies to refugees (including unaccompanied refugee minors), asylees, Amerasians, Afghan and Iraqi Special Immigrant Visa holders, and Cuban and Haitian parolees. Certified victims of human trafficking are also eligible for refugee benefits, including domestic medical screening. Spouses and dependent children of adults with humanitarian-based immigration status are also eligible to receive a domestic medical screening. *See screening recommendations^19^ and further information on the CDC website. Confirmation of *T. cruzi* infection requires positive results on at least two different tests based on different principles.

## References

[1] U.S. Department of Health and Human Services , Centers for Disease Control and Prevention , 2021. *Guidance for the U.S. Domestic Medical Examination for Newly Arriving Refugees*. Available at: https://www.cdc.gov/immigrantrefugeehealth/guidelines/domestic-guidelines.html. Accessed August 28, 2022.

[2] Lee BY Bacon KM Bottazzi ME Hotez PJ, 2013. Global economic burden of Chagas disease: a computational simulation model. Lancet Infect Dis 13: 342–348. 23395248 10.1016/S1473-3099(13)70002-1 PMC3763184

[3] Office of Immigration Statistics , 2020. *Refugees and Asylees: 2019. Annual Flow Report*. Available at: https://www.dhs.gov/sites/default/files/publications/immigration-statistics/yearbook/2019/refugee_and_asylee_2019.pdf. Accessed April 4, 2022.

[4] Luis Hassan Gallardo JB, 2020. *Venezuelan Immigrants in the United States*. Migration Policy Institute. Available at: https://www.migrationpolicy.org/article/venezuelan-immigrants-united-states-2018 Accessed April 20, 2022.

[5] Center for Disaster Philanthropy, 2022. *Venezuelan Humanitarian and Refugee Crisis*. Available at: https://disasterphilanthropy.org/disasters/venezuelan-refugee-crisis/. Accessed April 25, 2022.

[6] The UN Refugee Agency, n.d. *Displacement in Central America*. Available at: https://www.unhcr.org/en-us/displacement-in-central-america.html. Accessed April 25, 2022.

[7] Agudelo-Higuita N , 2022. U.S. bound journey of migrant peoples in transit across Dante’s Inferno and Purgatory in the Americas. Travel Med Infect Dis 47: 102317. 35342009 10.1016/j.tmaid.2022.102317

[8] Lucero RH , 2016. Chagas’ disease in Aboriginal and Creole communities from the Gran Chaco Region of Argentina: seroprevalence and molecular parasitological characterization. Infect Genet Evol 41: 84–92. 27057620 10.1016/j.meegid.2016.03.028

[9] Gabster A , 2021. Rapid health evaluation in migrant peoples in transit through Darien, Panama: protocol for a multimethod qualitative and quantitative study. Ther Adv Infect Dis 8: 20499361211066190. 34925828 10.1177/20499361211066190 PMC8679050

[10] Arnal A Waleckx E Rico-Chavez O Herrera C Dumonteil E, 2019. Estimating the current burden of Chagas disease in Mexico: a systematic review and meta-analysis of epidemiological surveys from 2006 to 2017. PLoS Negl Trop Dis 13: e0006859. 30964871 10.1371/journal.pntd.0006859 PMC6474657

[11] Conners EE Ordonez TL Cordon-Rosales C Casanueva CF Miranda SM Brouwer KC, 2017. Chagas disease infection among migrants at the Mexico/Guatemala border. Am J Trop Med Hyg 97: 1134–1140. 29016286 10.4269/ajtmh.16-0777 PMC5637586

[12] Montes-Rincon LM Galaviz-Silva L Molina-Garza ZJ, 2018. Anti-Trypanosoma cruzi antibodies in Latin American migrants in transit through the Mexico-USA border. Biomedica 38: 54–60. 29668134 10.7705/biomedica.v38i0.3526

[13] Velasco M , 2020. Screening for *Trypanosoma cruzi* infection in immigrants and refugees: systematic review and recommendations from the Spanish Society of Infectious Diseases and Clinical Microbiology. Euro Surveill 25: 1900393. 32127121 10.2807/1560-7917.ES.2020.25.8.1900393 PMC7055039

[14] Winters N Mora Izaguirre C, 2019. Es cosa suya: entanglements of border externalization and African transit migration in northern Costa Rica. CMS 7: 27.

[15] Vogt W, 2016. Stuck in the middle with you: the intimate labours of mobility and smuggling along Mexico’s migrant route. Geopolitics 21: 366–386.

[16] Yates C, 2019. *As More Migrants from Africa and Asia Arrive in Latin America, Governments Seek Orderly and Controlled Pathways*. Available at: https://www.migrationpolicy.org/article/extracontinental-migrants-latin-america. Accessed April 21, 2022.

[17] Carruth L Martinez C Smith L Donato K Pinones-Rivera C Quesada J, Migration Health in Social Context Working Group , 2021. Structural vulnerability: migration and health in social context. BMJ Glob Health 6 *(Suppl1):* e005109. 10.1136/bmjgh-2021-005109 PMC8031011 33827797

[18] Rodriguez F , 2021. Surveillance of *Trypanosoma cruzi* infection in triatomine vectors, feral dogs and cats, and wild animals in and around El Paso County, Texas, and New Mexico. PLoS Negl Trop Dis 15: e0009147. 33600455 10.1371/journal.pntd.0009147 PMC7924784

[19] Forsyth CJ , 2022. Recommendations for screening and diagnosis of Chagas disease in the United States. J Infect Dis. *225:* 1601–1610. 10.1093/infdis/jiab513 PMC9071346 34623435

[20] Villamil-Gomez WE Echeverria LE Ayala MS Munoz L Mejia L Eyes-Escalante M Venegas-Hermosilla J Rodriguez-Morales AJ, 2017. Orally transmitted acute Chagas disease in domestic travelers in Colombia. J Infect Public Health 10: 244–246. 27256221 10.1016/j.jiph.2016.05.002

[21] Carter YL Juliano JJ Montgomery SP Qvarnstrom Y, 2012. Acute Chagas disease in a returning traveler. Am J Trop Med Hyg 87: 1038–1040. 23091192 10.4269/ajtmh.2012.12-0354 PMC3516071

[22] Coura JR Vinas PA, 2010. Chagas disease: a new worldwide challenge. Nature 465: S6–S7. 20571554 10.1038/nature09221

[23] Coura JR, 2015. The main sceneries of Chagas disease transmission: the vectors, blood and oral transmissions—a comprehensive review. Mem Inst Oswaldo Cruz 110: 277–282. 25466622 10.1590/0074-0276140362 PMC4489464

[24] de Noya BA Gonzalez ON, 2015. An ecological overview on the factors that drives to *Trypanosoma cruzi* oral transmission. Acta Trop 151: 94–102. 26066984 10.1016/j.actatropica.2015.06.004

[25] Franco-Paredes C Villamil-Gomez WE Schultz J Henao-Martinez AF Parra-Henao G Rassi A Jr Rodriguez-Morales AJ Suarez JA, 2020. A deadly feast: elucidating the burden of orally acquired acute Chagas disease in Latin America: public health and travel medicine importance. Travel Med Infect Dis 36: 101565. 32004732 10.1016/j.tmaid.2020.101565

[26] Noya BA Diaz-Bello Z Colmenares C Ruiz-Guevara R Mauriello L Munoz-Calderon A Noya O, 2015. Update on oral Chagas disease outbreaks in Venezuela: epidemiological, clinical and diagnostic approaches. Mem Inst Oswaldo Cruz 110: 377–386. 25946155 10.1590/0074-02760140285 PMC4489475

[27] World Health Organization , 2015. Chagas disease in Latin America: an epidemiological update based on 2010 estimates. Wkly Epidemiol Rec 90: 33–43. 25671846

[28] Colombo V Giacomelli A Casazza G Galimberti L Bonazzetti C Sabaini F Ridolfo AL Antinori S, 2021. *Trypanosoma cruzi* infection in Latin American pregnant women living outside endemic countries and frequency of congenital transmission: a systematic review and meta-analysis. J Travel Med 28: taaa170. 32946555 10.1093/jtm/taaa170

[29] Centers for Disease Control and Prevention , 2012. Congenital transmission of Chagas disease: Virginia, 2010. MMWR Morb Mortal Wkly Rep 61: 477–479. 22763884

[30] Alarcon A Morgan M Montgomery SP Scavo L Wong EC Hahn A Jantausch B, 2016. Diagnosis and treatment of congenital Chagas disease in a premature infant. J Pediatr Infect Dis Soc 5: e28–e31. 10.1093/jpids/piw043 PMC10172994 27466398

[31] Bern C Montgomery SP, 2009. An estimate of the burden of Chagas disease in the United States. Clin Infect Dis 49: e52–e54. 19640226 10.1086/605091

[32] Kratz JM Garcia Bournissen F Forsyth CJ Sosa-Estani S, 2018. Clinical and pharmacological profile of benznidazole for treatment of Chagas disease. Expert Rev Clin Pharmacol 11: 943–957. 30111183 10.1080/17512433.2018.1509704

[33] Chadalawada S , 2020. Risk of chronic cardiomyopathy among patients with the acute phase or indeterminate form of Chagas disease: a systematic review and meta-analysis. JAMA Netw Open 3: e2015072. 32865573 10.1001/jamanetworkopen.2020.15072 PMC7489816

[34] Carlier Y Altcheh J Angheben A Freilij H Luquetti AO Schijman AG Segovia M Wagner N Albajar Vinas P, 2019. Congenital Chagas disease: updated recommendations for prevention, diagnosis, treatment, and follow-up of newborns and siblings, girls, women of childbearing age, and pregnant women. PLoS Negl Trop Dis 13: e0007694. 31647811 10.1371/journal.pntd.0007694 PMC6812740

[35] Perez-Zetune V Bialek SR Montgomery SP Stillwaggon E, 2020. Congenital Chagas disease in the United States: the effect of commercially priced benznidazole on costs and benefits of maternal screening. Am J Trop Med Hyg 102: 1086–1089. 32100696 10.4269/ajtmh.20-0005 PMC7204569

[36] Manne-Goehler J Umeh CA Montgomery SP Wirtz VJ, 2016. Estimating the burden of Chagas disease in the United States. PLoS Negl Trop Dis 10: e0005033. 27820837 10.1371/journal.pntd.0005033 PMC5098725

[37] Manne-Goehler J Reich MR Wirtz VJ, 2015. Access to care for Chagas disease in the United States: a health systems analysis. Am J Trop Med Hyg 93: 108–113. 25986581 10.4269/ajtmh.14-0826 PMC4497880

[38] Forsyth C Meymandi S Moss I Cone J Cohen R Batista C, 2019. Proposed multidimensional framework for understanding Chagas disease healthcare barriers in the United States. PLoS Negl Trop Dis 13: e0007447. 31557155 10.1371/journal.pntd.0007447 PMC6762052

[39] Forsyth CJ Stigler Granados P Pacheco GJ Betancourt JA Meymandi SK, 2019. Current gaps and needs for increasing access to healthcare for people with Chagas disease in the USA. Curr Trop Med Rep 6: 13–22.

[40] World Health Organization , 2020. Ending the Neglect to Attain the Sustainable Development Goals: A Road Map for Neglected Tropical Diseases 2021–2030. Geneva, Switzerland: World Health Organization.

